# Potential role of human umbilical cord stem cells-derived exosomes as novel molecular inhibitors of hepatocellular carcinoma growth

**DOI:** 10.1007/s10495-023-01863-z

**Published:** 2023-06-20

**Authors:** Hala M ElBadre, Sahar E M El-Deek, Haidi Karam-Allah Ramadan, Mohamed M Elbadr, Dina Sabry, Noran M Ahmed, Amr M Ahmed, Reham I El-Mahdy

**Affiliations:** 1 Department of Medical Biochemistry and Molecular Biology, Faculty of Medicine, Assiut University, Assiut, Egypt; 2 Department of Tropical Medicine and Gastroenterology, Faculty of Medicine, Assiut University, Assiut, Egypt; 3 Department of Medical Pharmacology, Faculty of Medicine, Assiut University, Assiut, Egypt; 4 Department of Medical Biochemistry and Molecular Biology, Faculty of Medicine, Badr University in Cairo, Badr City, Egypt; 5 Department of Medical Biochemistry and Molecular Biology, Faculty of Medicine, Cairo University, Cairo, Egypt; 6 Faculty of Medicine, Assiut University, Assiut, Egypt; 7 Department of Basic medical science, Badr University, west of Assiut, New Naser City, Assiut, Egypt

**Keywords:** Apoptosis, CXCR-4, HCC, Proliferation, SDF-1, Umbilical cord mesenchymal stem cells exosomes

## Abstract

Hepatocellular carcinoma (HCC) is one of the most critical cancers; thus, novel therapeutical regimens are of great need. In this study, we investigated the effects of umbilical cord mesenchymal stem cells (UC-MSCs) derived exosomes on HepG2 cell line, and the underlying mechanism to control HCC proliferation, to identify the potential clinical role of exosomes as a novel molecular therapeutic target. Proliferation, apoptosis, and angiogenesis effects were assessed together with the cell viability evaluation by MTT assay in HepG2 cells at 24/48 h. with or without UC-MSCs-derived exosomes. Gene expressions of TNF-α, caspase-3, VEGF, stromal cell-derived factor-1 (SDF-1), and CX chemokine receptor-4 (CXCR-4) were measured by quantitative real-time PCR technique. Expression of sirtuin-1 (SIRT-1) protein was detected by western blot. Treatment of HepG2 cells with UC-MSCs-derived exosomes for 24 and 48 h. demonstrated a significant reduction of cells survival compared to the control group (p < 0.05). The SIRT-1 protein, and VEGF, SDF-1, CXCR-4 expression levels were significantly lower, TNF-α and caspase-3 expression levels were significantly higher in exosomal-treated HepG2 cells for 24 and 48 h. than those in the control group. Moreover, our findings documented that the anti-proliferative, apoptotic, and anti-angiogenic effects were achieved in a time-dependent manner in which more effects were determined after 48 h supplementation compared to 24 h (p < 0.05). UC-MSCs-derived exosomes exert anticarcinogenic molecular effects on HepG2 cells through the involvement of SIRT-1, SDF-1, and CXCR-4. Hence, exosomes would be a potential novel therapy regimen against HCC. Large-scale studies are recommended to verify this conclusion.

**Supplementary Information:**

The online version contains supplementary material available at 10.1007/s10495-023-01863-z.

## Introduction

Exosomes are secreted by multiple cell types as extracellular vesicles containing active components, including lipids, proteins, and nucleic acids such as RNA and DNA [1]. They are secreted primarily in animals by the immune cells such as lymphocytes, platelets, dendritic cells, or red blood cells, and tumor cells [2]. These vesicles regulate cellular communication and play an important role in the diagnosis and development of many illnesses [1]. On the other side, exosomes can also affect the tumor microenvironment (TME) indirectly which regulates oncogenesis [3, 4].

Tumor-secreted exosomes then enhance angiogenesis in tumor tissues and play an important role in immune evasion. They can desensitize tumor cells to anti-cancer drugs inducing drug resistance. Finally, tumor derived-exosomes provide signals to the microenvironment activating the Epithelial-Mesenchymal Transition (EMT) which allows the progression of the tumor by invading the surrounding tissues and entering the circulation [5, 6]. Moreover, exosomes can be used as a non-invasive diagnostic method for tumors [7] because the expression of proteins and RNA in exosomes is specific to certain tissues and cell types [8]. Exosomes can also be modified to act as a vehicle for the delivery of therapeutic agents [6].

On the other hand, mesenchymal stem cells (MSCs) -derived exosomes have been reported to have beneficial effects in several animal models of liver diseases such as drug-induced liver injury, liver fibrosis, and hepatocellular carcinoma (HCC) [9–11]. MSCs-derived exosomes were shown to block the malignant behaviors of HCC stem cells [12].

Despite the advances in the treatment of HCC, it has a poor prognosis and a high risk of recurrence. HCC is the fifth most lethal cancer and the second cause of global cancer-related mortality [6]. Thus, it is fundamental to understand the molecular pathways involved in HCC development and progression, and to find novel non-invasive biomarkers for early diagnosis, and novel therapeutic targets for management.

The molecular pathogenesis of HCC results from genetic and epigenetic changes that are affected by the TME with subsequent different molecular and immune subtypes [13–15]. These molecular alterations include gene overexpression, or mutation, epigenetic silencing, and telomerase reactivation [16]. Exosomes have a major role in the development of HCC, via remodeling of TME, cell invasion, and angiogenesis. Moreover, they can carry specific functional molecules to increase the sensitivity to cancer drugs [17, 18].

In HCC, exosomes provide biologically active molecules in different stages of HCC progression, so they have the potential as diagnostic biomarkers as well as therapeutic targets [19, 20]. Therefore, analysis of this exosomal cargoes plays a vital role in demonstrating the biology of HCC, and identifying the direct targets of them as a potential therapeutic regimen for HCC [6].

Exosomes have attractive features, such as low immunogenicity, high biocompatibility, overcoming biological barriers [21], widely available, and are stable in most body fluids [1, 22]. Despite that Western blotting can be used to detect the content of characteristic proteins on the outer membrane of exosomes [17], the difficulty in isolation, purification, or synthetic technology of exosomes makes the studies on the effect on HCC inhibition by exosomes is still not clear enough. Subsequently, the diagnosis and treatment of exosomes related to HCC are still in the preclinical experimental stage.

Therefore, the aim of this study was to identify the potential role of exosomes as a novel molecular therapeutic target on HCC cell lines, and to study the underlying mechanism to control HCC proliferation.

## Materials and methods

The study procedures were approved by the Ethics Committee, Faculty of Medicine, Assiut University (Ethical Committee N: IRB 17300941) and had been performed according to the guidelines of the declaration of Helsinki.

### Preparation, isolation, and characterization of human umbilical cord mesenchymal stem cells (UC-MSCs)

After taking the parents’ informed consent, fresh human umbilical cord samples were obtained during caesarean deliveries of healthy newborn infants for healthy mothers. Wharton jelly was retrieved at the moment of birth from term deliveries. Collagenase II enzyme (IgG, C. histoliticum, Biological Life Science, USA) was used to isolate UC-MSCs, which were then digested and maintained in 2% foetal bovine serum and 1x Pen/Strep (Invitrogen, CA, USA). Cells were then incubated at 37° C, 5% CO2 till cells will reach 70–80% confluence, rinsed with phosphate buffer saline (PBS), and trypsinized at 37° C with 0.25% trypsin-EDTA (Invitrogen) for 5 min. After ultracentrifugation, cell pellets precipitate were resuspended with medium and maintained as first-passage cultures [23]. Every tube contained 1 × 10^5^ cells were mixed with 10 µL of monoclonal antibodies against the surface markers: mesenchymal surface markers CD 29 PE, CD34 PE, and CD 90 PE (Beckman coulter, USA) in the dark at 4° C. The same species isotypes were used as a negative control. After incubation, 2 ml of PBS containing 2% fetal calf serum (FCS) solution were supplied, centrifuged and cells were recovered with fresh medium. Cell evaluation was done using CYTOMICS FC 500 Flow Cytometer (Beckman coulter, FL, USA), and CXP Software version 2.2 was used for analysis.

### Isolation and identification of human UC-MSCs exosomes

Exosomes were liberated in conditioned media of UC-MSCs third passage (5 × 10^6^cells /ml) grown in RPMI devoid of FBS and enriched with 0.5% of bovine serum albumin (Sigma Aldrich). After centrifugation at 300 g, 2000 g, and 10,000 g for 20 min to eliminate debris, the larger cells, cell fragments, and dead cells. In some researches, filtration can replace these low-speed centrifugal steps for the large-scale preparation of exosomes (but this was not done in our work). The cell-free supernatant was then ultracentrifuged twice at 4 °C (Beckman Coulter Optima L 90 K ultracentrifuge) for 1 h at 100,000 x g to yield exosomes. Exosomes were washed in serum-free medium with HEPES buffer 25 mM (Sigma Aldrich) and submitted to a second ultracentrifugation under the same circumstances. After washing, a Fluorescence-Activated Cell Sorting, Calibur flow cytometer was used to analyze the obtained exosomes (Becton Dickinson, FACS Calibur). The analysis showed that they expressed CD44, CD29, α4- and α5 integrins in addition to CD73, but not α6-integrin [24]. Exosomes were stored at − 80 °C for future use in the experiment. The protein content of the extracted exosomes was determined by a Bradford protein analytical kit. The injection dose of exosomes was 100 µg protein/suspended in 0.2 ml PBS [25].

### Human hepatocellular carcinoma (HepG2) cell line

Human hepatocellular carcinoma cells (HepG2) as hepatic cancer cell line was purchased from VACSERA-Cell Culture Unit, Cairo, Egypt, which had previously got these cell lines from the American Type Culture Collection (ATCC, Minnesota, USA), it was seeded in Dulbecco’s Modified Eagle’s Medium (DMEM) and supplemented with 10% (v/v) fetal bovine serum and 1% (v/v) concentration ratio of penicillin and streptomycin (10,000 units penicillin and 10 mg/mL of streptomycin) (Lonza, Verviers, Belgium, cat. no. DE17-602E). HepG2 cells were grown in 50 cm^2^ culture flask and maintained in a 37 °C typical humidified incubator containing 5% CO2 and 95% air. Cells were washed with cold PBS, trypsinized, harvested in a falcon tube, and centrifuged to create cell pellets. The cultured HepG2 were divided into 4 groups as follows:

Group I (control untreated HepG2 24 h.): The HepG2 cells received no treatment for 24 h.

Group II (control untreated HepG2 48 h.): The HepG2 cells received no treatment for 48 h.

Group III (HepG2/exosomes 24 h.): The HepG2 cells were treated with human UC-MSC exosomes (100 µg/mL) for 24 h.

Group IV (HepG2/exosomes 48 h.): The HepG2 cells were treated with human UC-MSC exosomes (100 µg/mL) for 48 h.

### Determination of cytotoxicity effect by MTT assay

The anti-proliferative effect of UC-MSC exosomes on HepG2 cells, was explored using 3-(4,5-dimethylthiazol-2-yl)-2,5-diphenyltetrazolium bromide (MTT) assay. The HepG2 cells of the four studied groups were fixed at a frequency of 5000 cells into 96 well flat-bottom plates for 24 h. After that, the cells were treated with UC-MSC exosomes. Human UC-MSC exosomes were added to the HepG2cell monolayer for 24, and 48 h at 37 ◦C. MTT reagent was added to the wells in line with the manufacturer’s guidelines (Sigma-Aldrich Co.) and composite to the entire wells plate for 4 to 6 h. When the purple precipitate was obviously visible, a detergent reagent was supplemented (100 µl per well) to allow the formazan dye soluble. Plates were placed with cover in the dim for 2 to 4 h. The plate cover was removed and the resulted color change was assessed spectrophotometrically at a range from 490 to 630 nm. Readings were taken and the average was calculated. Half maximal inhibitory concentration (IC50) values of UC-MSC exosomes were measured using the Prism software version 5.0 (GraphPad Software Inc., San Diego, CA, USA).

### Quantitative real‑time polymerase chain reaction (qRT-PCR)

The effect of human UC-MSC exosomes on gene expression was evaluated using quantitative real-time PCR. HepG2 cells up to 1 × 10^5^ cell/well were cultured in a 6 well plate at IC50 concentration of UC-MSC exosomes. Cells were rinsed with ice-cold PBS, trypsinized, collected, centrifuged, and lysed in 200 µl cold RNA lysis buffer containing 5 µl RNase (20 µg/ml) for 15 min.

Total purified RNA was isolated using Thermo Fisher Scientific Inc. Germany (GeneJET, Kit, #K0732) following the manufacturer’s guidelines. The yield of the resultant total RNA was estimated at 260 and 280 nm by using Nanodrop® (Epoch Microplate Spectrophotometer, Biotek, USA). The total RNA of each sample was reverse transcribed to the complementary DNA using the High-Capacity cDNA synthesis kit (Applied Biosystems, CA, USA). PCR amplification cycles using Maxima SYBR Green qPCR master mix kit (Thermo Fisher Scientific, USA, Catalog #K0251) were done.

Reactions were run using real-time PCR (Step one 7500 fast, Applied biosystem, Foster city, USA). Subsequently, 40 cycles of amplification were performed, which involved DNA denaturation at 95 °C for 15 s, primers annealing at 55 °C for 20 s, and at 72 °C for 30 s. The data were expressed in cycle threshold (CT) following the RT-PCR run. The relative expression of each target gene is calculated by normalization against the mean CT values of the 18 s RNA housekeeping gene using the ΔΔCt method [26]. The primers sequences for each gene used in this study are listed in Table 1.

**Table 1:** The sequence of primers used for RT-PCR analysis of mRNA expression

Target gene	Primer sequence (5′-3′) (F: Forward primer), (R: Reverse primer)	Gene bank accession number	Product length	Tm
TNF-α	F: CTCTTCTGCCTGCTGCACTTTG R: ATGGGCTACAGGCTTGTCACTC	NM_000594.4	135	62
Caspase-3	F: TGACAGCCAGTGAGACTTGG R: GACTCTAGACGGCATCCAGC	XM_047416239.1	112	59
VEGF	F: CGGGAACCAGATCTCTCACC R: AAAATGGCGAATCCAATTCC	NM_003376.5	233	59
CXCR4	F: CTGAGAAGCATGACGGACAA R: TCGATGCTGATCCCAATGTA	XM_047445802.1	581	57
SDF-1	F: AGAGCCAACGTCAAGCATCT R: GGGCAGCCTTTCTCTTCTTC	NM_001033886.2	223	59
18s RNA	F: CAGCCACCCGAGATTGAGCA R: TAGTAGCGACGGGCGGGTG	JX_132355.1	244	61

### Western blot analysis

HepG2 cells were lysed with RIPA lysis buffer, and proteins from the entire cell lysate were obtained. A total of 30 µg of exosomes protein were separated by 7% SDS PAGE on polyacrylamide gradient gels (TGX Stain-Free™ FastCast™ Acrylamide kit). After incubation in 5% non-fat dried milk dissolved in Tris-buffered saline-Tween 20 (TBST) buffer for 1 h., primary antibodies; rabbit anti-Sirtuin-1 (1:500 dilution) were applied to Western blotting membranes, PVDF loaded with specimen samples and incubated overnight at 4 °C. At room temperature, mouse anti-β-actin (1:1000 dilution) (Santa Cruz Biotechnology, Santa Cruz, CA, USA) was incubated for 1 h. After being washed twice with TBST buffer, quantification of the Western blot bands was done using Image J software on the ChemiDoc MP imaging system made by Bio-Rad (Hercules, CA). Relative density of immunoblot was assessed and normalized with β-actin (27).

All procedures were carried out at the Medical Biochemistry and the Molecular Biology Research Center, Assiut University, Egypt and Biochemistry Unit, Faculty of Medicine, Cairo University.

### Statistical analysis

Data were examined using SPSS version 20 (SPSS Inc., Chicago, IL, USA) and GraphPad Prism 5 software (San Diego, California, USA). All variables were analyzed before evaluation to determine if variables were parametric or not by the normality Kolmogorov– Smirnov test (KS) and Shapiro–Wilk test. The mean, and standard deviation (SD) were considered in quantitative data. Comparisons between continuous variables were performed using the Mann–Whitney test and Student’s t-test. For clarification of results, P values were taken to be considered as statistically significant if less than 0.05.

## Results

### Characterization of human UC-MSCs and exosomes

**Fig. 1:**
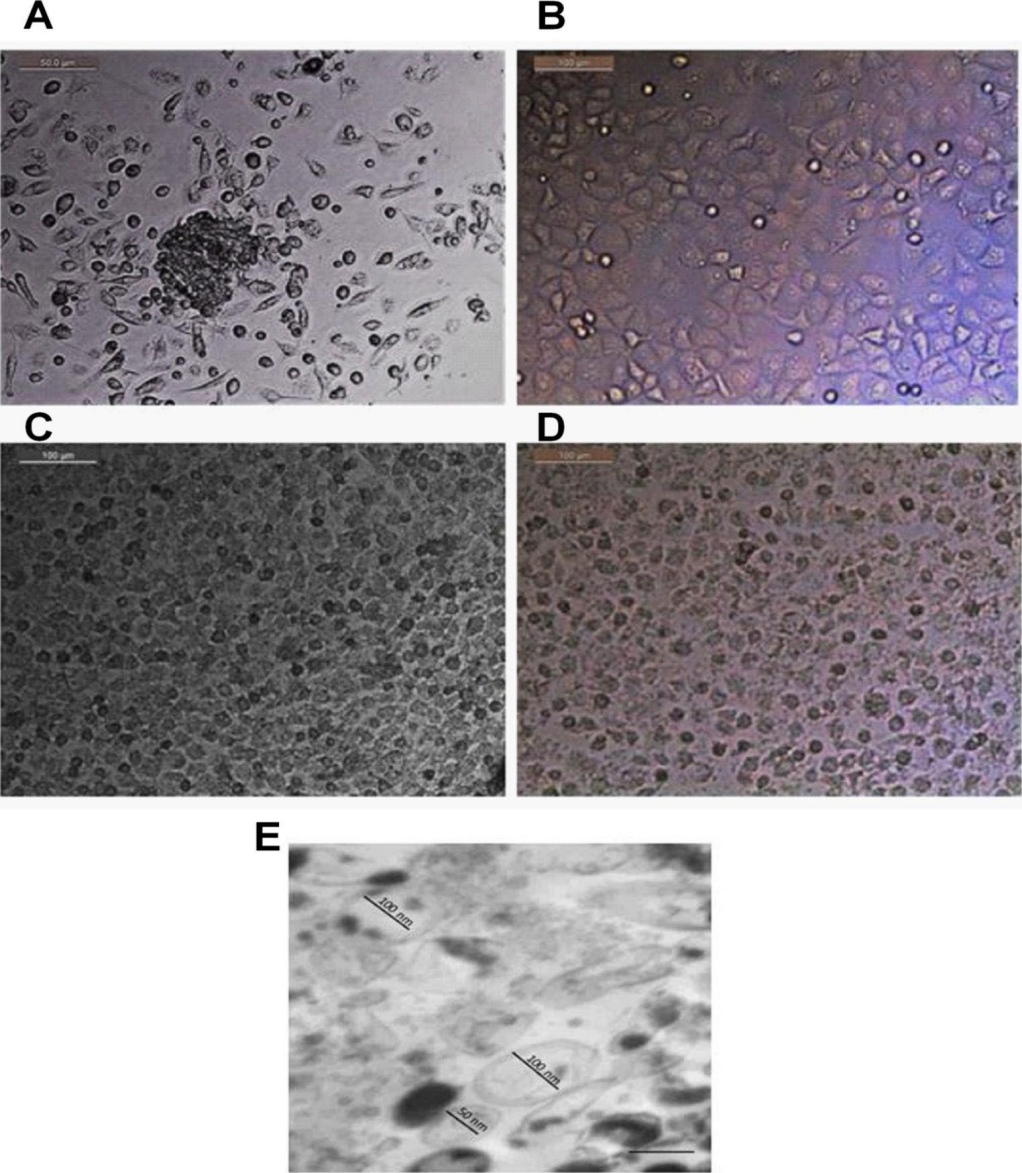
**Microscopic cell morphology of cultured mesenchymal stem cells.** (**A**): Human hematopotiec derived stem cells (scale bar, 50 μm). (**B**): Human hepatocellular carcinoma cell line (HepG2) (scale bar, 100 μm). (**C**): HepG2/exosomes for 24 h (scale bar, 100 μm). (**D**): HepG2/exosomes for 48 h (scale bar, 100 μm). (**E**): TEM analysis showed variable sizes of Exosomes, Scale bar 2 μm

MSCs were recognized by their morphology, where MSCs were distinguished by their fibroblast fusiform shape, elliptical nucleus, and colony-forming unit. Phenotypical characterization of exosomes was detected by their size (100 nm) and by their cup-shaped spheroidal morphology (Fig. 1).

### Effect of UC-MSCs exosomes on the viability of HepG2 cells

**Fig. 2:**
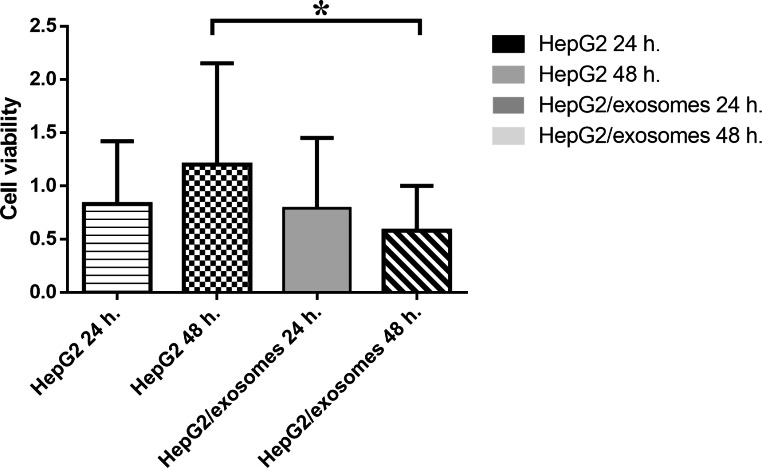
Human umbilical cord mesenchymal stem cells-derived exosomes inhibit cell proliferation in HepG2 cells. The cell viability was evaluated at different studied groups by MTT assay proliferation kit. Data show mean ± SD values. ∗p < 0.05

To analyze the ability of UC-MSCs exosomes to inhibit hepatocellular cancer cell viability, HepG2cells were treated with UC-MSCs exosomes, and the cell viability was evaluated after 24 and 48 h. of treatment using the MTT assay. As shown in Fig. 2, and Table 2, UC-MSCs exosomes significantly decreased cancer cell viability where UC-MSCs exosomes treatment for 48 h. (p < 0.05) were much more potent than UC-MSCs exosomes treatment for 24 h. and cause strong significant growth inhibition in the HCC cell line. This suggests that UC-MSCs exosomes have an anti-proliferative effect on HepG2 cells in a time-dependent manner.

**Table 2:** Comparison between different studied groups regarding cell viability

Cell viability	HepG2 24 h	HepG2 48 h	HepG2/exosomes 24 h	HepG2/exosomes 48 h
Mean ± SD	0.83 ± 0.59 ^b ns c ns d ns^	1.2 ± 0.95 ^ans cns d*^	0.79 ± 0.66 ^a ns bns d ns^	0.58 ± 0.42 ^ans b*c ns^
Median (IQR)	0.51(1.04)	0.84(1.65)	0.58(0.98)	0.38(0.54)

a: comparison with the HepG2 24 h,

b: comparison with the HepG2 48 h,

c: comparison with the HepG2/exosomes 24 h,

d: comparison with the HepG2/exosomes 48 h

∗p < 0.05, ns: non-significant

IQR: Interquartile range

### Effect of UC-MSCs exosomes on the cell proliferation

**Fig. 3:**
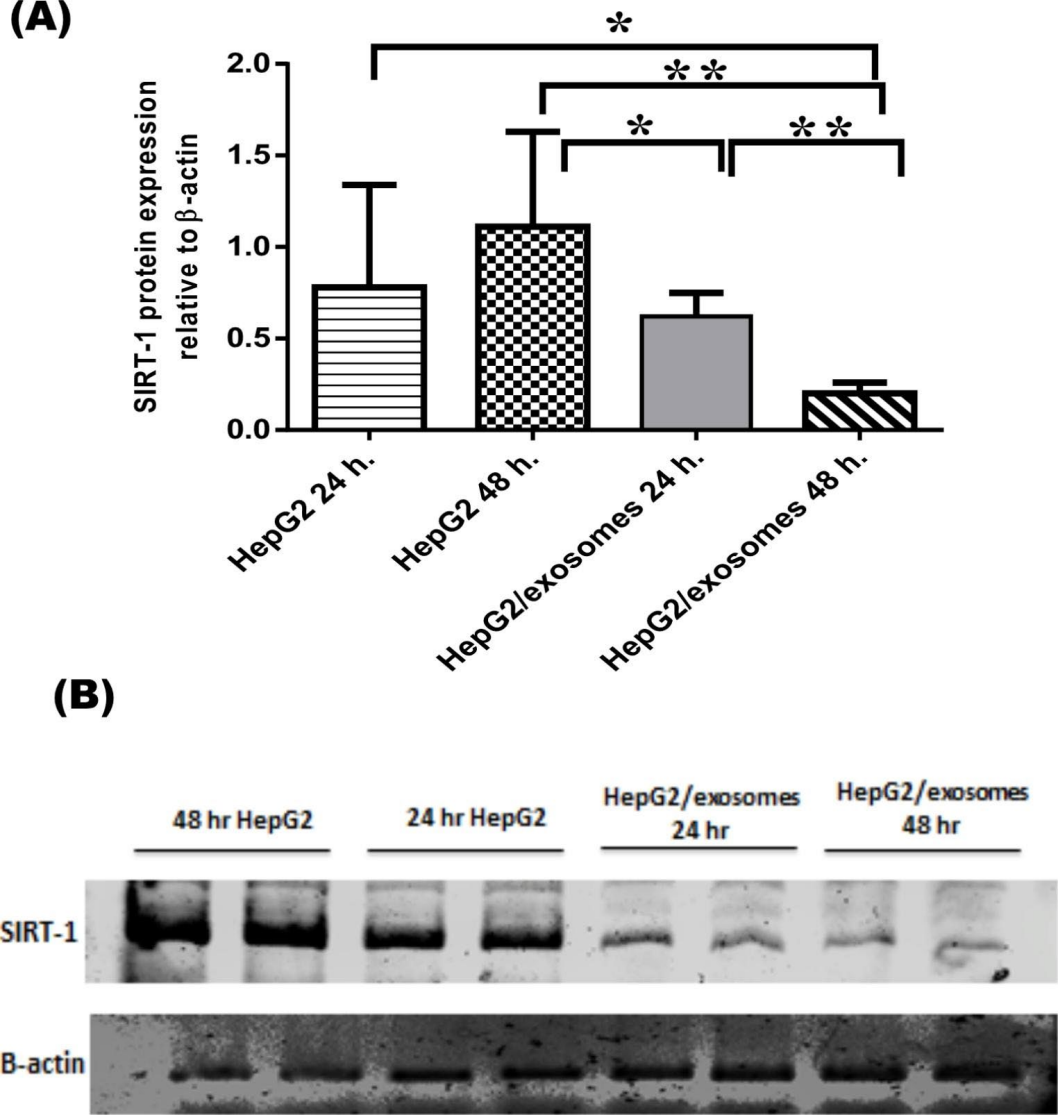
The effect of human umbilical cord mesenchymal stem cells-derived exosomes on sirtuin-1 level (by western blot) in HepG2 cells. (**A**): Results are expressed as means ± SD among the different studied groups, ∗p < 0.05, ∗∗p < 0.01. (**B**): The scanning densitometry western blot of SIRT-1 relative to housekeeping protein β-actin in all studied groups

To further investigate the mechanism underlying the anti-proliferative effects of UC-MSCs exosomes on the HCC cell line, we assessed Sirtuin-1 (SIRT-1) expression levels in HepG2 cells using the western blot technique. SIRT-1 demonstrated lower expression levels in HepG2 cells treated with UC-MSCs exosomes for 24 h. (0.62 ± 0.13) compared to non-treated HepG2cells for 24 h. (0.78 ± 0.56) and 48 h. (1.11 ± 0.52). Also, SIRT-1 decreased significantly on HepG2 cells treatment with UC-MSCs exosomes for 48 h. (0.20 ± 0.06) in comparison to their corresponding levels in non-treated HepG2 cells for 24 h. (0.78 ± 0.56), HepG2 cells for 48 h. (1.11 ± 0.52), and HepG2/exosomes 24 h. (0.62 ± 0.13) (Fig. 3).

### Effect of UC-MSCs exosomes on apoptosis

**Fig. 4:**
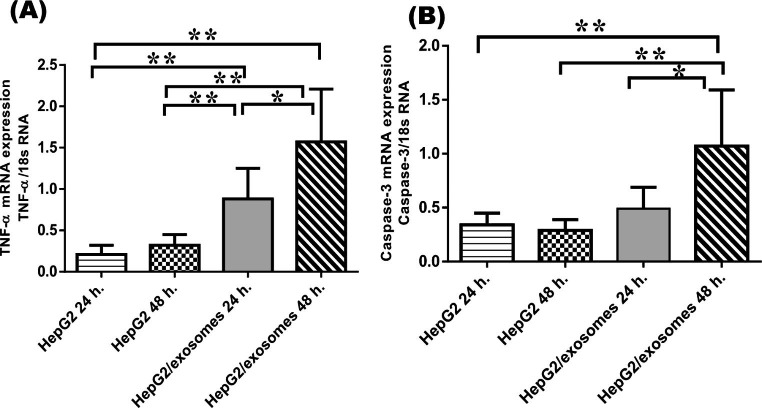
Expression levels of the studied apoptotic markers in HepG2 cells (**A**): Relative quantitative expression of mRNA level of TNF-α. (**B**): Relative quantitative expression of mRNA level of caspase-3. Gene expression level was normalized to 18s RNA. Results are expressed as means ± SD among the different studied groups. p value is considered significant when < 0.05, ∗p < 0.05, ∗∗p < 0.01

Treatment of HepG2cells with UC-MSCs exosomes caused significantly increased relative expression mean levels of tumor necrosis factor-alpha (TNF-α) in HepG2 cells treated with UC-MSCs exosomes for 24 h.(0.88 ± 0.37) compared to their corresponding levels in non-treated HepG2 cells for 24 h. (0.21 ± 0.11) and 48 h. (0.32 ± 0.13). Moreover, supplementation of the HepG2 cells with UC-MSCs exosomes for 48 h. resulted in a significant upregulation in their mean levels of TNF-α expression (1.57 ± 0.64) as compared to non-treated HepG2 cells for 24 h. (0.21 ± 0.11), HepG2 cells for 48 h. (0.32 ± 0.13), and HepG2/exosomes 24 h. (0.88 ± 0.37) (Fig. 4A).

Mean relative expression of caspase-3 was upregulated significantly in HepG2 cells treated with UC-MSCs exosomes for 48 h. (1.07 ± 0.52) compared to HepG2 24 h. (0.34 ± 0.11), HepG2 48 h.(0.29 ± 0.10), and HepG2/exosomes 24 h. (0.49 ± 0.20) (Fig. 4B).

### Effect of UC-MSCs exosomes on angiogenesis

**Fig. 5:**
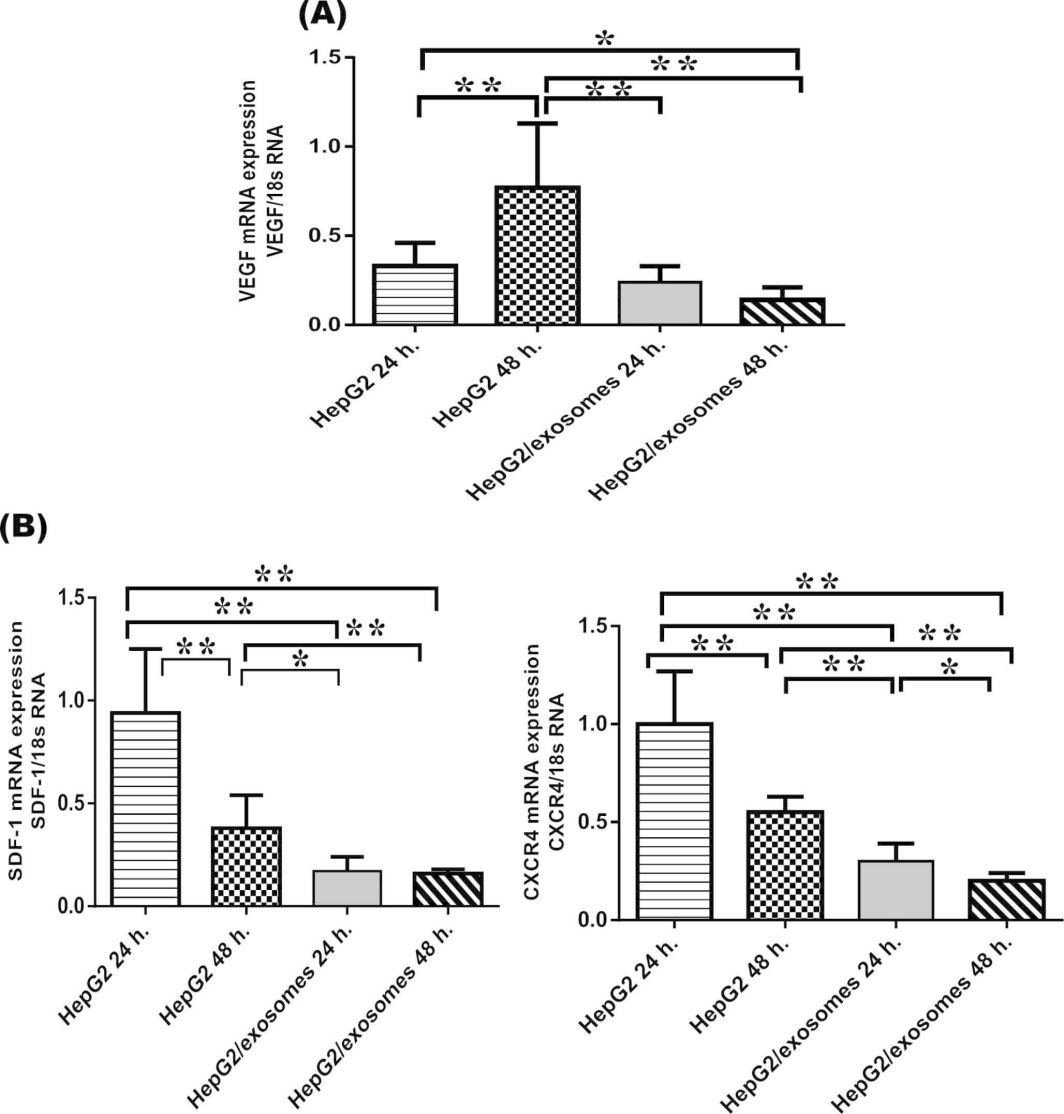
Expression levels of the studied angiogenic markers in HepG2 cells (**A**): Relative quantitative expression of mRNA level of vascular enothelial growth factor (VEGF). (**B**): Relative quantitative expression of mRNA level of stromal cell-derived factor-1 (SDF-1) or C-X-C motif chemokine (CXCl-12) and CX chemokine receptor-4 (CXCR-4). Gene expression level was normalized to 18s RNA. Results are expressed as means ± SD among the different studied groups. p value is considered significant when < 0.05, ∗p < 0.05, ∗∗p < 0.01

Vascular endothelial growth factor (VEGF) mean relative expression was reduced significantly in the HCC cell line treated with UC-MSCs exosomes for 24 h. (0.24 ± 0.09) (p < 0.01) in contrast to non-treated cells for 48 h. (0.77 ± 0.36). In addition, the cells treated with UC-MSCs exosomes for 48 h. resulted in significant decrease in VEGF expression level (0.14 ± 0.07) compared to HepG2 24 h.(0.24 ± 0.09), and HepG2 48 h. (0.77 ± 0.36) (Fig. 5A).

Regarding stromal cell-derived factor-1 (SDF-1) or C-X-C motif chemokine (CXCl-12) and CX chemokine receptor-4 (CXCR-4), relative quantitative expression of mRNA level analyses results were shown in Fig. 5. UC-MSCs exosomes treatment for 24 h. caused a significant decline in the mean expression of SDF-1 and CXCR-4 levels (0.17 ± 0.07, and 0.30 ± 0.09, respectively) as compared to the control untreated HepG2 cells (HepG2 24 h.(0.94 ± 0.31, 1 ± 0.27, respectively), and HepG2 48 h. (0.38 ± 0.16, 0.55 ± 0.08, respectively). Moreover, HepG2 cells treated with UC-MSCs exosomes for 48 h. demonstrated a significant time-dependent downregulation in SDF-1 (0.16 ± 0.02) and CXCR-4 (0.20 ± 0.04) mRNA expression levels in comparison to the control untreated HepG2 cells (p < 0.01) and exosomes treatment for 24 h. (p < 0.05 for CXCR-4).

## Discussion

Pathogenesis of HCC is a multistep process including genetic and epigenetic alterations in the context of the liver microenvironment [28]. External stimuli such as hepatitis B or C viruses, alcohol or aflatoxin induce alteration to the hepatocytes or stem cells [29, 30], initiating the process of apoptosis, cellular proliferation, dysplasia, and neoplasia [31].

Exosomal cargos in HCC trigger a cascade of signaling in the recipient cells, facilitating oncogenesis, angiogenesis, tumor proliferation, and metastasis [19]. On the other hand, the tumor suppressor found in the exosomes can directly display tumor suppression effects. For example, exosomal miR-122 produced by HCC can inhibit EMT, increase drug sensitivity, and inhibit angiogenesis [32, 33].

In the current study, the viability and proliferation of the HCC cell line (HepG2) were lowered following the addition of exosomes after 24 and 48 h. That could be explained by the analysis of the measured parameters of cell proliferation, apoptosis, and angiogenesis in the study.

SIRT-1 as a marker of proliferation was downregulated in HepG2/exosomes after 24 h, and was significantly lower after 48 h. SIRT1 is a NAD+-dependent enzyme class-III histone deacetylase (HDAC) and its down-regulation was described in HCC. Sirtuins are involved in the prevention of DNA damage and enhancing DNA repair by many different pathways. They are involved in gene regulation, genome stability, apoptosis, autophagy, proliferation, senescence, and oncogenesis.

However, SIRT1 can act as either a tumor suppressor or a tumor promoter [34]. This explains its anti-proliferative effect in this study, as SIRT1 can modulate the activity of proteins necessary for oncogenesis and enhance tumor proliferation and progression as well as drug resistance [35, 36].

Regarding the measured parameters of apoptosis in the current study, TNF-α and caspase-3 level was high in HepG2/exosomes after 24 h relative to HepG2 cells and further increased in HepG2/exosomes after 48 h. This is similar to the study conducted by Bruno et al. who reported the effect of bone marrow-MSCs on the induction of apoptosis in HepG2 cells [37].

Apoptosis has a vital role in protecting the host against tumor development by enhancing the elimination of DNA-damaged cells [38]. Two main apoptotic pathways exist: the extrinsic or receptor-mediated pathway, and the intrinsic or mitochondrial pathway [39]. The extrinsic pathway is activated when a receptor of the tumor necrosis factor receptor superfamily (TNFRSF) binds with its corresponding death ligand (TNFSF). The intrinsic pathway is activated by oxygen free radicals, and interleukin (IL)-1 and IL-6. Both pathways activate caspase-3, which is the main executor of cell death [40, 41].

TNF-α as a major pro-inflammatory cytokine is usually involved in tumor proliferation, and metastasis [42]. However, the accumulation of a high level of TNF-α in the tumor tissues exhibits potent anti-neoplastic actions [43] which were in concordance with this study. Moreover, the high level of TNF-α in HepG2/exosome induces the extrinsic pathways of HCC cell lines apoptosis which activated caspase-3, the principal enzyme in the apoptotic cascade used to detect apoptotic activity [44].

The angiogenic markers in this study either VEGF, CXCL12, or CXCR4 showed a significant low level in HepG2/exosomes after 24 h and a significant lower level after 48 h. CXCL12, known as SDF- 1, is the only specific endogenous ligand for CXCR4 [45].

Endogenous regulators of angiogenesis include a wide range and heterogenous group of factors and the major factor is vascular endothelial growth factor A (VEGF-A) [46].VEGF induces vascular permeability, which enhances the spread of tumor cells into the bloodstream and promotes distant metastases [47].

Another factor in the angiogenesis is CXCR4 and its ligand CXCL12, which by activating the MAPK/ERK and PI3K/Akt signaling pathways, initiate cell migration and angiogenesis. Also by inducing the expression of matrix metalloproteinase 10 (MMP10) in HCC cells via the ERK1/2 pathway, promote HCC cells growth, proliferation, and metastasis [48, 49]. Additionally, by binding to CXCR4, CXCL12 participates in cell chemotaxis, migration, proliferation, and differentiation [50, 51]. CXCL12 also plays a major role in the recruitment of Treg cells into TME which contribute to the growth and spread of HCC [52]. Hence, CXCR4 and its ligand provide potential targets for the treatment of liver diseases, and tumors [53, 54].

Exosomes, by providing several pro-angiogenic and anti-angiogenic factors such as mRNA, miRNA, and proteins, induce changes in the functional profile of the recipient cells [55]. Therefore, in the current study, down-regulation of VEGF, CXCL12, and CXCR4 induced by UC-MSCs exosomes reduced the angiogenesis of HepG2 HCC cell lines which reduced their proliferative capacity at the end. Similar to our study, Lee et al. showed that the addition of MSC-derived exosomes decreased VEGF secretion in breast cancer cells, consistent with the down-regulation of VEGF mRNA levels and miR-16 [56].

The overall results of this study indicate the role of exosomes in regulating the dynamic cross-talk between different cell populations in TME limiting HCC cell line growth and spread. This is consistent with Pan et al. who reported that MSC-derived exosomes can inhibit HCC development by transfection with miRNAs [57].

This provides an important base for the strategies of anti-tumor target therapy. However, further in profundity studies are still needed to determine which factors drive the cross-talk mediated by exosomes toward changing the functional profile of the recipient cells into the proliferative or anti-proliferative pathways and the downstream signaling ligands of SIRT-1, SDF-1, and CXCR-4.

## Conclusion

This is the first study to show that UC-MSCs-derived exosomes exerted anti-proliferative apoptotic, and anti-angiogenic effects against HCC due to the suppression of SIRT-1 protein expression, the upregulation of TNF-α and caspase-3 expressions as well as the downregulation of VEGF, SDF-1 and CXCR-4 expressions. The findings of the current study strongly suggested that UC-MSCs-derived exosomes could be an effective novel molecular therapy against HCC cells in vitro. These results may help to improve the prognosis of HCC.

## Electronic supplementary material

Below is the link to the electronic supplementary material.

Supplementary Material 1

## Data Availability

All related data and materials are available from the corresponding author upon request.
