# Supplementary figures and images for: Potential role of human umbilical cord stem cells-derived exosomes as novel molecular inhibitors of hepatocellular carcinoma growth
Source: Apoptosis. 2023 Jun 20;28(9-10):1346–56. doi: 10.1007/s10495-023-01863-z (PMC10425301; doi:10.1007/s10495-023-01863-z)

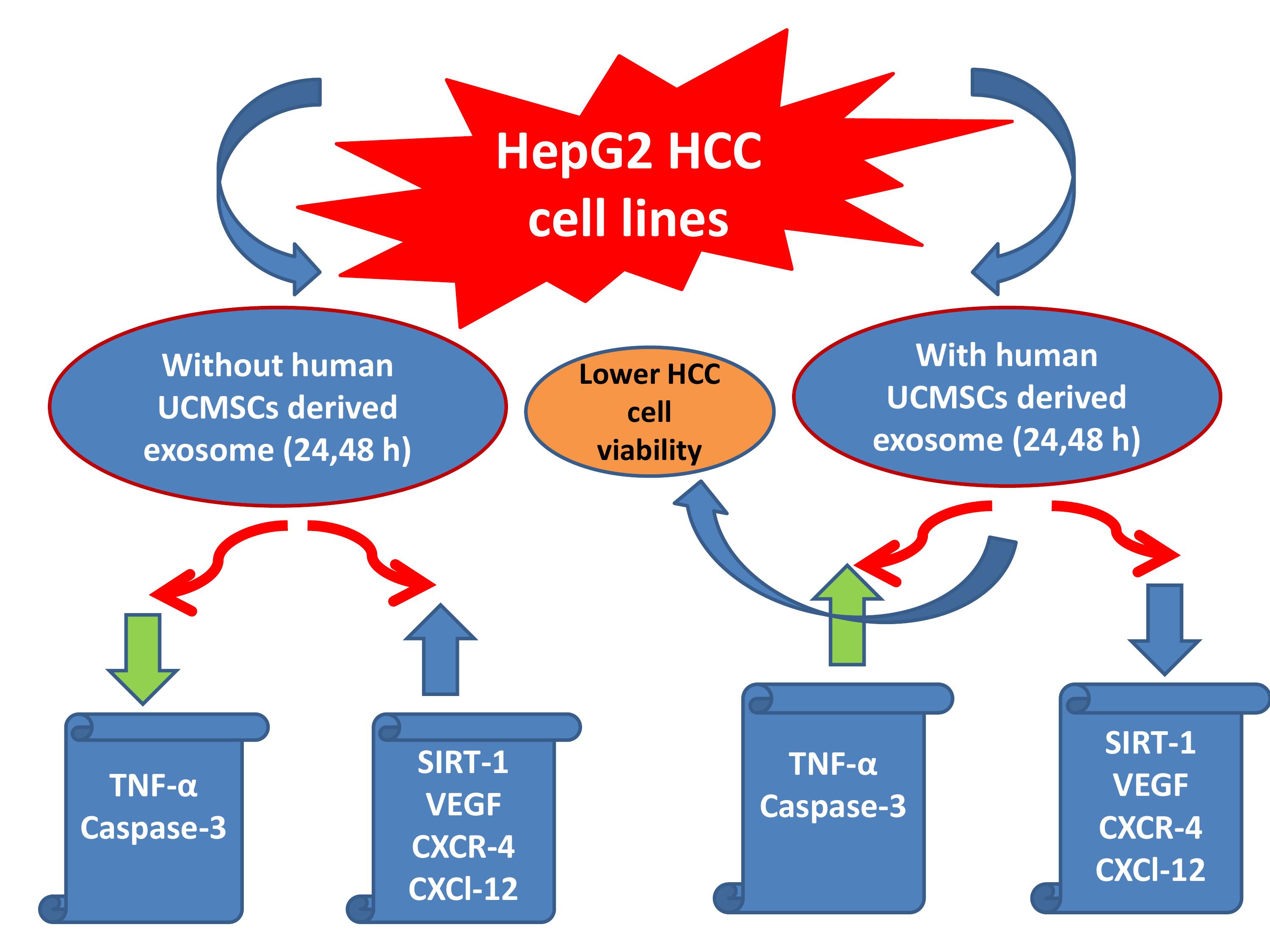

Supplement: Supplementary file 1 — Supplementary Material 1 [file 10495_2023_1863_MOESM1_ESM.jpg]
